# Modeling and Optimizing Medium Composition for Shoot Regeneration of Chrysanthemum via Radial Basis Function-Non-dominated Sorting Genetic Algorithm-II (RBF-NSGAII)

**DOI:** 10.1038/s41598-019-54257-0

**Published:** 2019-12-03

**Authors:** Mohsen Hesami, Roohangiz Naderi, Masoud Tohidfar

**Affiliations:** 1 Department of Horticultural Science, Faculty of Agriculture, University of Tehran, Karaj, Iran; 2 Department of Plant Biotechnology, Faculty of Science and Biotechnology, Shahid Beheshti University, Tehran, Iran

**Keywords:** Agricultural genetics, Computational models

## Abstract

The aim of the current study was modeling and optimizing medium compositions for shoot proliferation of chrysanthemum, as a case study, through radial basis function- non-dominated sorting genetic algorithm-II (RBF-NSGAII). RBF as one of the artificial neural networks (ANNs) was used for modeling four outputs including proliferation rate (PR), shoot number (SN), shoot length (SL), and basal callus weight (BCW) based on four variables including 6-benzylaminopurine (BAP), indole-3-butyric acid (IBA), phloroglucinol (PG), and sucrose. Afterward, models were linked to the optimization algorithm. Also, sensitivity analysis was applied for evaluating the importance of each input. The R^2^ correlation values of 0.88, 0.91, 0.97, and 0.76 between observed and predicted data were obtained for PR, SN, SL, and BCW, respectively. According to RBF-NSGAII, optimal PR (98.85%), SN (13.32), SL (4.83 cm), and BCW (0.08 g) can be obtained from a medium containing 2.16 µM BAP, 0.14 µM IBA, 0.29 mM PG, and 87.63 mM sucrose. The results of sensitivity analysis indicated that PR, SN, and SL were more sensitive to BAP, followed by sucrose, PG, and IBA. Finally, the performance of predicted and optimized medium compositions were tested, and results showed that the difference between the validation data and RBF-NSGAII predicted and optimized data were negligible. Generally, RBF-NSGAII can be considered as an efficient computational strategy for modeling and optimizing *in vitro* organogenesis.

## Introduction

Chrysanthemum (*Dendranthema* × *grandiflorum* Ramat.) is known as one of the most important ornamental plants, which has worldwide values as cut flowers, herbaceous landscape plants, and flowering potted plants due to its wide range of color, shape, structure, and size^1^. Shoot proliferation of chrysanthemum could be one of the useful methods for commercial and high quality/price propagation in the nursery and cut-flower industries as well as breeding programs^2^. Moreover, genetic engineering by using biolistic or *Agrobacterium* methods could be considered as a viable alternative to traditional breeding methods for developing unique chrysanthemum cultivars in order to satisfy market demand^3^. However, transformation efficiencies in chrysanthemum, obtained by these methods, remain low because of the lack of efficiency of *in vitro* organogenesis procedures^2,3^. Pavingerová, *et al*.^4^ reported that high applicability of transformation in chrysanthemum is completely depended on the organogenesis procedures. Therefore, the use of *in vitro* culture for producing chrysanthemum could reduce the frequency of problems occurring in the commercial production of this plant^1–4^.

Adjusting the culture medium concentrations of plant growth regulators (PGRs)^5^, sucrose, and phloroglucinol (PG)^6^ could enhance the propagation potential of different genotypes and explants^7,8^. Thus, it is significant to improve the organogenesis protocols by using suitable components in order to overcome difficulties associated with clonal regeneration and gene transformation strategies^9–11^.

PGRs play an important role in *in vitro* organogenesis such as shoots regeneration, somatic embryogenesis, and callus induction^12,13^. The required concentrations of PGRs in plant tissue culture may vary from species to species, while the cytokinin/auxin ratio plays an essential role in *in vitro* culture^14–16^.

Since explants are not photosynthetically active during the primary step of *in vitro* culture, carbohydrate is an essential component for *in vitro* organogenesis by supplying the carbon content and maintaining the osmotic potential of cells^17^. Sucrose, glucose, and fructose are three types of common carbohydrates in plant tissue culture^18^. Sucrose is broadly applied in *in vitro* culture because of its positive effects on plant growth and development and low cost. However, the concentration of sucrose and its interaction with other medium compositions are very important for successful *in vitro* organogenesis^17^.

PG is a phenolic compound known to contribute to different plant developmental processes^19^. The exterior usage of PG might improve the tolerance of plants under various biotic stresses^19^. In recent years, PG was implicated as beneficial to *in vitro* plant propagation^19–25^. Sarkar and Naik^22^ indicated that phloroglucinol could enhance the regeneration response through increasing the number of meristems in potato and showed that PG × sucrose interaction plays an important role in shoot regeneration. Also, Steephen, *et al*.^23^ have shown that PG exerts a powerful impact on *in vitro* organogenesis and proliferation. Siwach and Gill^24^ and De Klerk, *et al*.^25^ showed that *in vitro* shoot proliferation as well as root formation of plantlets were promoted significantly by applying PG to the MS medium in *Ficus religiosa* and apple rootstocks, respectively. Although there are several studies about the effect of PG on improving *in vitro* organogenesis^19–25^, there is no research evidence on the effect of this molecule and its interaction with other medium components on shoot proliferation of chrysanthemum.

The traditional analytical approaches for the solving problems of *in vitro* culture are not suitable to model non-linear and non-deterministic systems such as *in vitro* organogenesis^26–31^. Nowadays, data-driven models such as fuzzy logic and neural networks have been proposed as suitable alternatives to model the ill-defined and non-linear systems^26–32^. Artificial neural networks (ANNs) could be useful in modeling and optimizing organogenesis during plant tissue culture^26,27,30^. In a modeling study, multilayer perceptron (MLP) was used to model the impacts of sucrose contents, nitrate concentrations, fresh weight, size, and type of explant per vessel, temperature, pH, time of inoculation, and the volume of growth medium in hairy root culture^33^. In another modeling study, MLP was applied for modeling *in vitro* sterilization of chrysanthemum rootstocks^34^. However, there is a lack of comprehensive studies regarding the effectiveness of the radial basis function (RBF) as an ANN method for modeling the compositions of the medium.

Recently, some studies applied a genetic algorithm (GA) for optimization to reduce computational volumes^26,27^. By using GA, as one of the well-known optimization algorithm, optimal solutions can be achieved with minimal computing. Also, *in vitro* culture problems have to overcome different objective functions through considering different constraints. For instance, basal callus has a negative effect on micropropagation due to the somaclonal variation and restriction of the vascular system. Therefore, the ultimate aim of shoot proliferation protocols would be the maximum proliferation rate and the minimum basal callus^2^. However, Multi-objective functions cannot be optimized by using a single- objective function such as GA^34–36^. Therefore, multi-objective algorithms are necessary for the optimization of outputs. Classical optimization methods, including multi-criterion decision making methods, have provided a model for converting multi-objective optimization to a single-objective optimization issue through emphasizing one particular Pareto-optimal solution at a time. This approach requires multiple runes in order to obtain different possible solutions^35^. One of the first evolutionary multi-objective optimization algorithms, which is useful for finding the solution domain in order to discover Pareto-optimal solutions within a multi-objective centered scheme is known as The Non-dominated Sorting Genetic Algorithm-II (NSGA-II)^37^.

In this study, we tried to propose a model for shoot proliferation by using RBF. We have linked the model to optimization algorithm (NSGAII) to find the maximum efficiency and the optimum medium compositions levels which are necessary for significant *in vitro* organogenesis. This study was aimed to use RBF- NSGAII for modeling and optimizing the appropriate medium compositions for shoot multiplication of chrysanthemum.

## Results

### Effects of medium composition on shoot proliferation

Although many studies have investigated the impact of PGRs levels in shoot proliferation of chrysanthemum, there is a lack of comprehensive investigation on the effect of medium compositions. Medium composition is a vital factor in *in vitro* culture that significantly affects shoot regeneration. In this study, the interaction effects of 6-benzylaminopurine (BAP), indole-3-butyric acid (IBA), PG, and sucrose on proliferation rate (PR), shoot number (SN), shoot length (SL), and basal callus weight (BCW) of chrysanthemum were studied.

The results indicated that higher PR, SN, SL, and BCW were achieved in MS medium supplemented with both BAP and IBA. However, no PR, SN, SL, and BCW were observed on MS medium without PGRs. By increasing the concentration of BAP (up to 2.22 µM), PR, SN, and SL were enhanced significantly. Also, in the absence of PG, the highest PR, SN, and SL were observed in the combination of 2.22 µM BAP and 0.25 µM IBA (Table 1). Furthermore, sucrose concentration had a significant effect on PR, SN, SL, and BCW. Thus, the highest PR, SN, SL, and BCW were observed in the 87.64 mM sucrose (Table 1). According to Table 1, the highest PR (100%), SN (12.76), and SL (4.6 cm) were achieved in MS medium supplemented with 2.22 µM BAP, 0.25 µM IBA, 0.2 mM PG, and 87.64 mM sucrose.

**Table 1:** Effects of BAP, IBA, PG, and sucrose concentrations on proliferation rate (PR), shoot number (SN), shoot length (SL), and basal callus weight (BCW) of chrysanthemum.

BAP (µM)	IBA (µM)	PG (mM)	Sucrose (mM)	PR (%)	SN	SL (cm)	BCW (g)
0	0	0	43.82	0.00 ± 0.00	0.00 ± 0.00	0.00 ± 0.00	0.00 ± 0.00
0	0.25	0	43.82	0.00 ± 0.00	0.00 ± 0.00	0.00 ± 0.00	0.00 ± 0.00
0	0.49	0	43.82	0.00 ± 0.00	0.00 ± 0.00	0.00 ± 0.00	0.00 ± 0.00
2.22	0	0	43.82	31.11 ± 3.51	1.33 ± 0.14	2.40 ± 0.13	0.00 ± 0.00
2.22	0.25	0	43.82	33.33 ± 3.33	1.56 ± 0.15	2.69 ± 0.15	0.00 ± 0.00
2.22	0.49	0	43.82	28.89 ± 3.51	1.39 ± 0.16	2.57 ± 0.17	0.00 ± 0.00
4.44	0	0	43.82	40.00 ± 5.77	1.53 ± 0.23	2.74 ± 0.17	0.04 ± 0.02
4.44	0.25	0	43.82	42.22 ± 5.21	1.72 ± 0.21	2.96 ± 0.18	0.10 ± 0.03
4.44	0.49	0	43.82	55.56 ± 2.94	1.87 ± 0.17	2.89 ± 0.15	0.16 ± 0.04
0	0	0.2	43.82	0.00 ± 0.00	0.00 ± 0.00	0.00 ± 0.00	0.00 ± 0.00
0	0.25	0.2	43.82	0.00 ± 0.00	0.00 ± 0.00	0.00 ± 0.00	0.00 ± 0.00
0	0.49	0.2	43.82	0.00 ± 0.00	0.00 ± 0.00	0.00 ± 0.00	0.00 ± 0.00
2.22	0	0.2	43.82	48.89 ± 4.84	1.80 ± 0.20	2.81 ± 0.16	0.00 ± 0.00
2.22	0.25	0.2	43.82	62.22 ± 5.21	2.83 ± 0.22	3.01 ± 0.16	0.00 ± 0.00
2.22	0.49	0.2	43.82	55.56 ± 4.44	2.56 ± 0.28	3.21 ± 0.19	0.00 ± 0.00
4.44	0	0.2	43.82	31.11 ± 3.51	1.33 ± 0.14	2.74 ± 0.17	0.08 ± 0.03
4.44	0.25	0.2	43.82	40.00 ± 4.71	2.07 ± 0.20	3.32 ± 0.14	0.16 ± 0.04
4.44	0.49	0.2	43.82	55.56 ± 4.44	3.07 ± 0.20	3.79 ± 0.15	0.21 ± 0.04
0	0	0.4	43.82	0.00 ± 0.00	0.00 ± 0.00	0.00 ± 0.00	0.00 ± 0.00
0	0.25	0.4	43.82	0.00 ± 0.00	0.00 ± 0.00	0.00 ± 0.00	0.00 ± 0.00
0	0.49	0.4	43.82	0.00 ± 0.00	0.00 ± 0.00	0.00 ± 0.00	0.00 ± 0.00
2.22	0	0.4	43.82	57.78 ± 5.21	2.66 ± 0.18	3.46 ± 0.14	0.00 ± 0.00
2.22	0.25	0.4	43.82	53.33 ± 6.67	2.76 ± 0.21	3.63 ± 0.14	0.00 ± 0.00
2.22	0.49	0.4	43.82	64.44 ± 5.56	3.64 ± 0.10	4.06 ± 0.11	0.00 ± 0.00
4.44	0	0.4	43.82	44.44 ± 4.44	2.26 ± 0.15	3.68 ± 0.15	0.13 ± 0.02
4.44	0.25	0.4	43.82	71.11 ± 3.51	3.66 ± 0.15	3.71 ± 0.13	0.22 ± 0.04
4.44	0.49	0.4	43.82	62.22 ± 4.01	3.41 ± 0.13	3.92 ± 0.15	0.28 ± 0.05
0	0	0	87.64	0.00 ± 0.00	0.00 ± 0.00	0.00 ± 0.00	0.00 ± 0.00
0	0.25	0	87.64	0.00 ± 0.00	0.00 ± 0.00	0.00 ± 0.00	0.00 ± 0.00
0	0.49	0	87.64	0.00 ± 0.00	0.00 ± 0.00	0.00 ± 0.00	0.00 ± 0.00
2.22	0	0	87.64	60.00 ± 4.71	5.52 ± 0.21	3.30 ± 0.12	0.00 ± 0.00
2.22	0.25	0	87.64	51.11 ± 4.84	5.43 ± 0.12	3.50 ± 0.10	0.04 ± 0.02
2.22	0.49	0	87.64	48.89 ± 3.51	3.10 ± 0.26	4.18 ± 0.17	0.09 ± 0.03
4.44	0	0	87.64	53.33 ± 5.77	2.80 ± 0.34	3.59 ± 0.11	0.10 ± 0.02
4.44	0.25	0	87.64	57.78 ± 5.21	2.63 ± 0.21	3.94 ± 0.09	0.10 ± 0.03
4.44	0.49	0	87.64	60.00 ± 5.77	3.09 ± 0.29	4.62 ± 0.12	0.17 ± 0.04
0	0	0.2	87.64	0.00 ± 0.00	0.00 ± 0.00	0.00 ± 0.00	0.00 ± 0.00
0	0.25	0.2	87.64	0.00 ± 0.00	0.00 ± 0.00	0.00 ± 0.00	0.00 ± 0.00
0	0.49	0.2	87.64	0.00 ± 0.00	0.00 ± 0.00	0.00 ± 0.00	0.00 ± 0.00
2.22	0	0.2	87.64	93.33 ± 3.33	8.82 ± 0.16	4.20 ± 0.15	0.00 ± 0.00
**2.22**	**0.25**	**0.2**	**87.64**	**100.00 **±** 0.00**	**12.76 **±** 0.25**	**4.60 **±** 0.14**	**0.07 **±** 0.02**
2.22	0.49	0.2	87.64	100.00 ± 0.00	11.46 ± 0.20	4.82 ± 0.13	0.07 ± 0.02
4.44	0	0.2	87.64	84.44 ± 4.44	6.76 ± 0.14	4.84 ± 0.15	0.11 ± 0.03
4.44	0.25	0.2	87.64	95.56 ± 2.94	8.19 ± 0.16	5.10 ± 0.09	0.19 ± 0.04
4.44	0.49	0.2	87.64	100.00 ± 0.00	9.10 ± 0.15	5.74 ± 0.11	0.24 ± 0.03
0	0	0.4	87.64	0.00 ± 0.00	0.00 ± 0.00	0.00 ± 0.00	0.00 ± 0.00
0	0.25	0.4	87.64	0.00 ± 0.00	0.00 ± 0.00	0.00 ± 0.00	0.00 ± 0.00
0	0.49	0.4	87.64	0.00 ± 0.00	0.00 ± 0.00	0.00 ± 0.00	0.00 ± 0.00
2.22	0	0.4	87.64	91.11 ± 3.51	8.62 ± 0.11	5.06 ± 0.12	0.00 ± 0.00
2.22	0.25	0.4	87.64	100.00 ± 0.00	11.43 ± 0.18	4.77 ± 0.17	0.13 ± 0.03
2.22	0.49	0.4	87.64	95.56 ± 2.94	9.76 ± 0.18	5.63 ± 0.13	0.19 ± 0.03
4.44	0	0.4	87.64	86.67 ± 3.33	6.39 ± 0.15	5.54 ± 0.17	0.21 ± 0.02
4.44	0.25	0.4	87.64	91.11 ± 3.51	7.87 ± 0.15	5.70 ± 0.13	0.32 ± 0.03
4.44	0.49	0.4	87.64	100.00 ± 0.00	8.59 ± 0.14	5.89 ± 0.13	0.40 ± 0.03
0	0	0	131.46	0.00 ± 0.00	0.00 ± 0.00	0.00 ± 0.00	0.00 ± 0.00
0	0.25	0	131.46	0.00 ± 0.00	0.00 ± 0.00	0.00 ± 0.00	0.00 ± 0.00
0	0.49	0	131.46	0.00 ± 0.00	0.00 ± 0.00	0.00 ± 0.00	0.00 ± 0.00
2.22	0	0	131.46	46.67 ± 3.33	4.04 ± 0.21	3.22 ± 0.14	0.00 ± 0.00
2.22	0.25	0	131.46	44.44 ± 2.94	5.04 ± 0.08	3.18 ± 0.09	0.03 ± 0.02
2.22	0.49	0	131.46	40.00 ± 3.33	2.51 ± 0.19	3.79 ± 0.17	0.08 ± 0.04
4.44	0	0	131.46	42.22 ± 5.21	2.33 ± 0.28	3.43 ± 0.10	0.06 ± 0.02
4.44	0.25	0	131.46	53.33 ± 4.71	2.32 ± 0.18	3.54 ± 0.14	0.08 ± 0.03
4.44	0.49	0	131.46	53.33 ± 4.71	2.39 ± 0.38	4.07 ± 0.12	0.12 ± 0.03
0	0	0.2	131.46	0.00 ± 0.00	0.00 ± 0.00	0.00 ± 0.00	0.00 ± 0.00
0	0.25	0.2	131.46	0.00 ± 0.00	0.00 ± 0.00	0.00 ± 0.00	0.00 ± 0.00
0	0.49	0.2	131.46	0.00 ± 0.00	0.00 ± 0.00	0.00 ± 0.00	0.00 ± 0.00
2.22	0	0.2	131.46	84.44 ± 4.44	8.40 ± 0.14	3.49 ± 0.09	0.00 ± 0.00
2.22	0.25	0.2	131.46	95.56 ± 2.94	10.76 ± 0.21	3.81 ± 0.16	0.03 ± 0.02
2.22	0.49	0.2	131.46	91.11 ± 3.51	9.09 ± 0.16	4.23 ± 0.08	0.07 ± 0.02
4.44	0	0.2	131.46	73.33 ± 4.71	5.68 ± 0.14	4.60 ± 0.13	0.09 ± 0.03
4.44	0.25	0.2	131.46	86.67 ± 4.71	7.61 ± 0.12	5.10 ± 0.09	0.17 ± 0.02
4.44	0.49	0.2	131.46	93.33 ± 3.33	8.59 ± 0.17	5.34 ± 0.12	0.19 ± 0.03
0	0	0.4	131.46	0.00 ± 0.00	0.00 ± 0.00	0.00 ± 0.00	0.00 ± 0.00
0	0.25	0.4	131.46	0.00 ± 0.00	0.00 ± 0.00	0.00 ± 0.00	0.00 ± 0.00
0	0.49	0.4	131.46	0.00 ± 0.00	0.00 ± 0.00	0.00 ± 0.00	0.00 ± 0.00
2.22	0	0.4	131.46	80.00 ± 3.33	6.76 ± 0.18	4.58 ± 0.15	0.00 ± 0.00
2.22	0.25	0.4	131.46	91.11 ± 3.51	9.57 ± 0.19	4.71 ± 0.10	0.09 ± 0.02
2.22	0.49	0.4	131.46	86.67 ± 4.71	8.49 ± 0.23	4.70 ± 0.19	0.14 ± 0.03
4.44	0	0.4	131.46	77.78 ± 5.21	5.61 ± 0.14	5.33 ± 0.15	0.13 ± 0.03
4.44	0.25	0.4	131.46	80.00 ± 4.71	7.11 ± 0.16	4.94 ± 0.23	0.21 ± 0.04
4.44	0.49	0.4	131.46	84.44 ± 5.56	7.59 ± 0.16	5.22 ± 0.24	0.27 ± 0.04

Values in each column represent means ± SE.

### RBF modeling and evaluation

RBF was used for modeling the four outputs (PR, SN, SL, and BCW) based on four variables, including BAP, IBA, PG, and sucrose.

**Figure 1:**
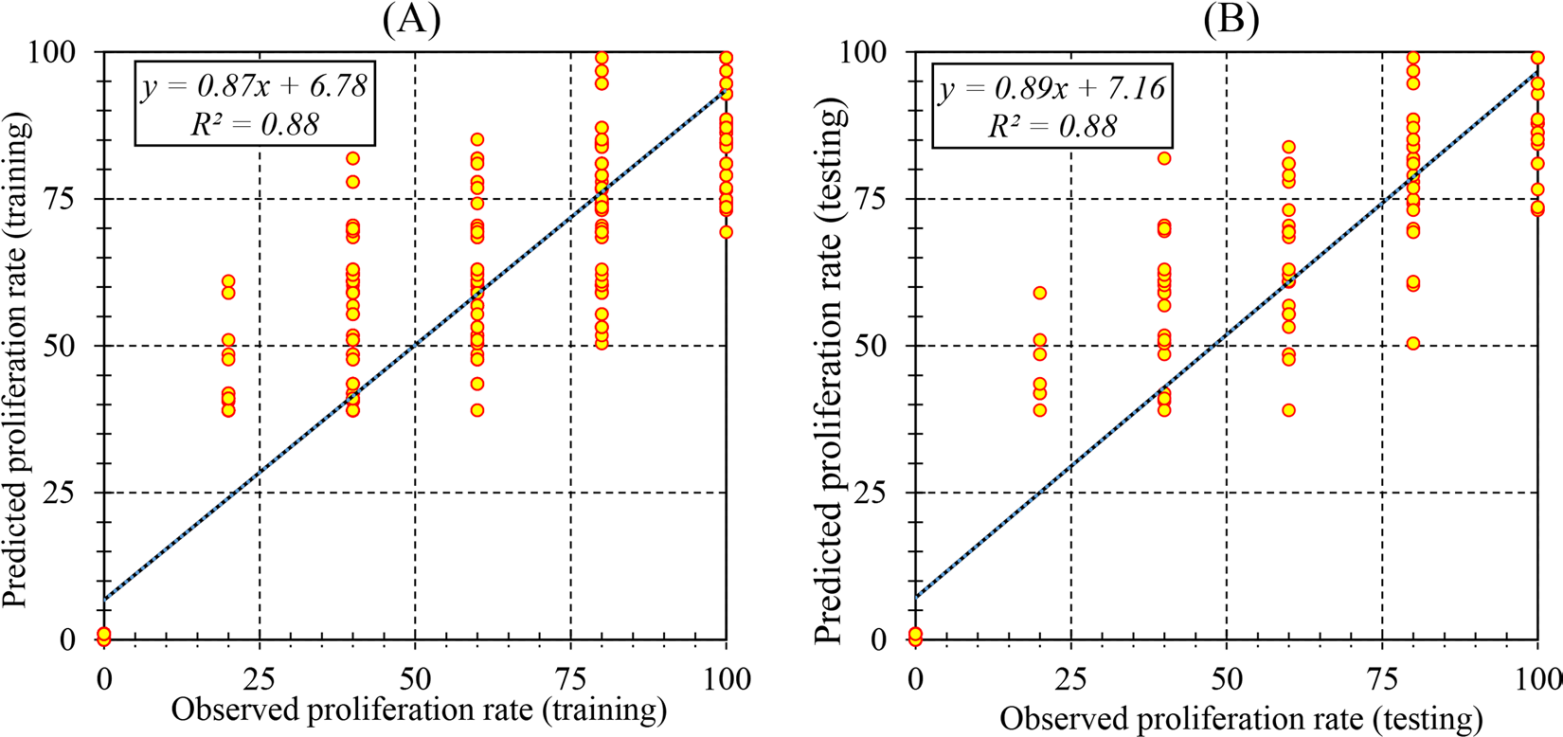
Scatter plot of model predicted vs. observed values of proliferation rate (PR) of chrysanthemum for medium composition formulation obtained by RBF model. (**A**) Training set (n = 547); (**B**) Testing set (n = 182). Fitted simple regression line on scatter points was indicated by a solid line.

**Figure 2:**
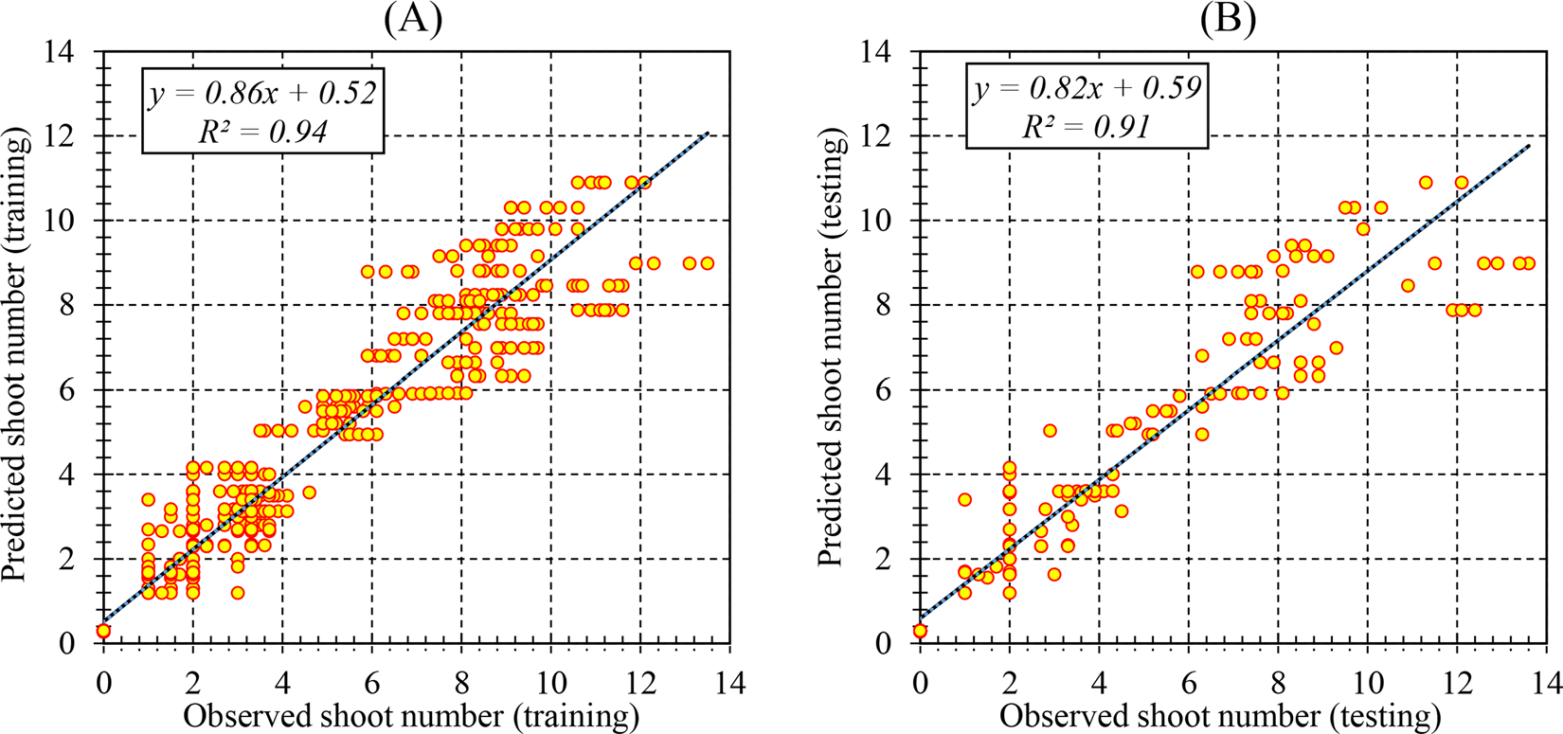
Scatter plot of model predicted vs. observed values of shoot number (SN) of chrysanthemum for medium composition formulation obtained by RBF model. (**A**) Training set (n = 547); (**B**) Testing set (n = 182). Fitted simple regression line on scatter points was indicated by a solid line.

**Figure 3:**
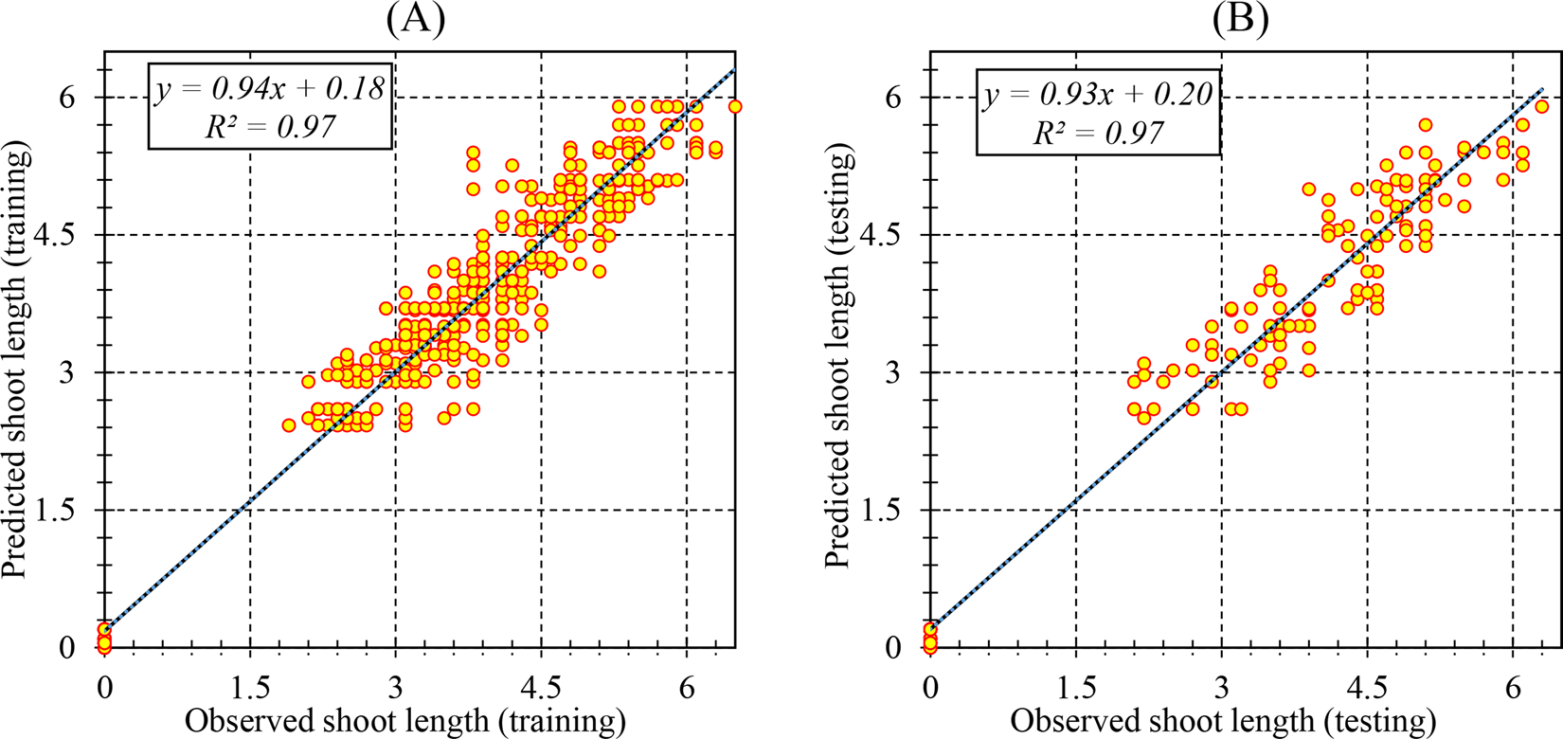
Scatter plot of model predicted vs. observed values of shoot length (SL) of chrysanthemum for medium composition formulation obtained by RBF model. (**A**) Training set (n = 547); (**B**) Testing set (n = 182). Fitted simple regression line on scatter points was indicated by a solid line.

**Figure 4:**
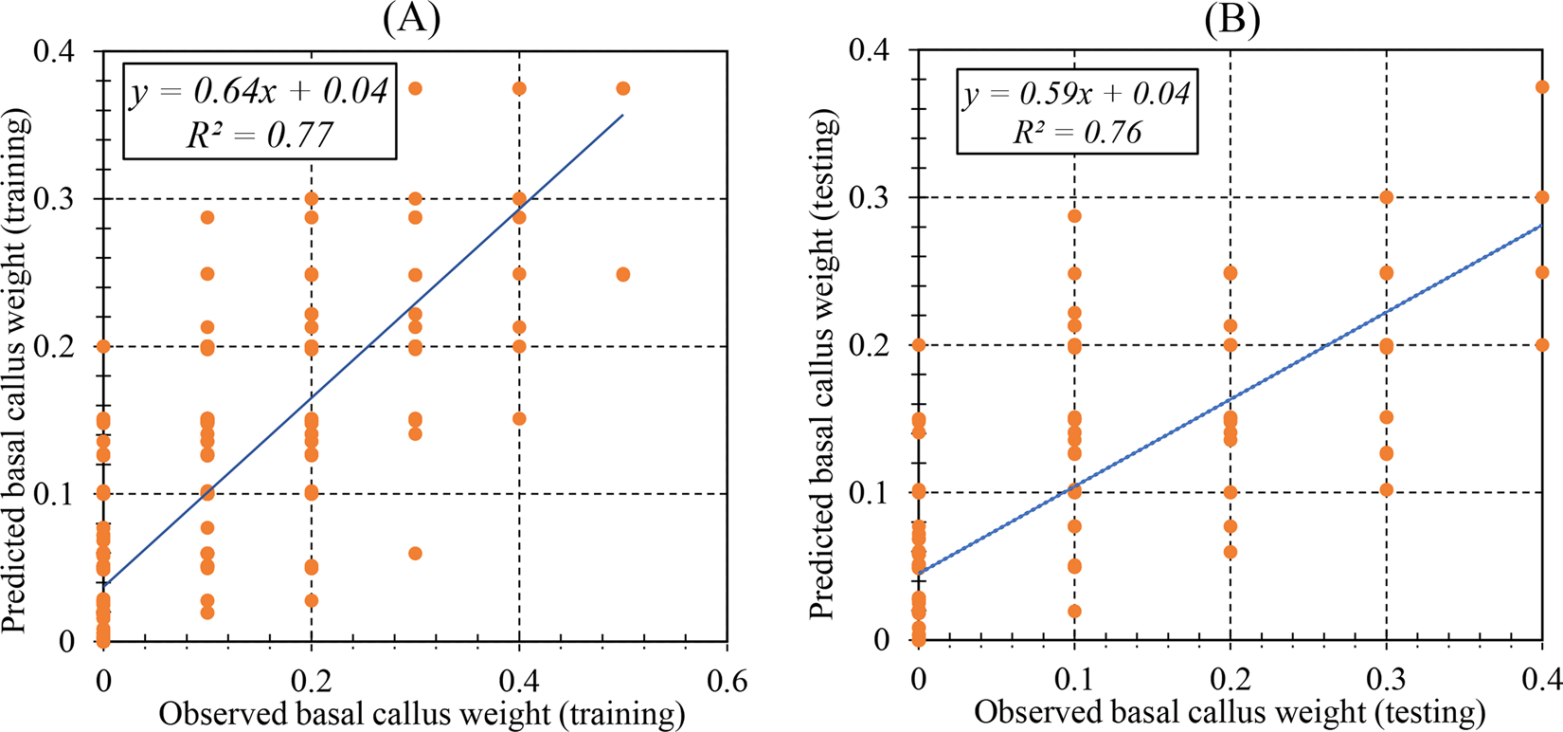
Scatter plot of model predicted vs. observed values of basal callus weight (BCW) of chrysanthemum for medium composition formulation obtained by RBF model. (**A**) Training set (n = 547); (**B**) Testing set (n = 182). Fitted simple regression line on scatter points was indicated by a solid line.

The efficiency of the RBF models was described based on the assessment of predicted and observed data on both the train and test set. The results (Table 2) showed that the RBF models were successful in predicting PR, SN, SL, and BCW with correlations between predicted and observed data showing a good fit (Figs. 1, 2, 3 and 4). Furthermore, similar performance of the RBF models was achieved on both training and testing data indicating the model did not over-fit.

**Table 2:** Statistics of RBF models for proliferation rate (PR), shoot number (SN), shoot length (SL), and basal callus weight (BCW) of chrysanthemum (training vs. testing values).

Item	PR		SN		SL		BCW	
	Training	Testing	Training	Testing	Training	Testing	Training	Testing
R^2^	0.88	0.88	0.94	0.91	0.97	0.97	0.77	0.76
RMSE	13.10	13.38	0.96	1.20	0.36	0.38	0.11	0.07
MBE	0.76	2.49	0.01	−0.06	0.03	0.02	0.01	0.02

### Model optimization

The ultimate aim of the current study was to optimize RBF model by NSGA-II for providing an accurate answer of what levels of medium compositions may be applied to obtain the maximum PR, SN, and SL as well as the minimum BCW. Thus, the model linked to NSGAII for finding the maximum efficiency and the optimum medium compositions levels, which are essential for significant *in vitro* shoot regeneration.

The RBF provides adequate accuracy for interpolation but not for extrapolation^38^. Therefore, the upper bound and lower bound of input variables (Table 1) were set as constraints, and the point with the highest PR, SN, and SL as well as the lowest BCW was considered as the ideal point during the optimization process. According to RBF-NSGA-II (Table 3), optimal PR (98.85%), SN (13.32), SL (4.83 cm), and BCW (0.08 g) can be obtained from a medium containing 2.16 µM BAP, 0.14 µM IBA, 0.29 mM PG and 87.63 mM sucrose.

**Table 3:** Optimizing medium composition according to optimization analysis on the developed RBF-NSGAII in the ideal point for proliferation rate (PR), shoot number (SN), shoot length (SL), and basal callus weight (BCW) in chrysanthemum.

Input variable				Predicted RF (%)	Predicted SN	Predicted SL (cm)	Predicted BCW (g)
BAP (µM)	IBA (µM)	PG (mM)	Sucrose (mM)				
2.16	0.14	0.29	87.63	98.85	13.32	4.83	0.08

### Sensitivity analysis of the models

The importance of each input was evaluated through the entire 729 data lines (training and testing) to determine the general VSR. The VSR achieved for the model output (PR, SN, SL, and BCW), with respect to medium compositions (Table 4). Sensitivity analysis showed that PR, SN, and SL were more sensitive to BAP, followed by sucrose, PG, and IBA (Table 4). In BCW model, the feed efficiency indicated more sensitivity for BAP, followed by IBA, PG, and sucrose (Table 4).

**Table 4:** Importance of medium composition for proliferation rate (PR), shoot number (SN), shoot length (SL), and basal callus weight (BCW) of chrysanthemum according to sensitivity analysis on the developed RBF model to rank the importance of inputs.

Output	Item	BAP	IBA	PG	Sucrose
PR	VSR	2.85	1.01	1.35	1.36
	Rank	1	4	3	2
SN	VSR	3.14	1.17	1.99	2.36
	Rank	1	4	3	2
SL	VSR	6.07	1.15	1.65	1.87
	Rank	1	4	3	2
BCW	VSR	1.34	1.09	1.06	1.05
	Rank	1	2	3	4

### Validation experiment

The results of the validation experiment (Table 5) showed that RBF-NSGAII could be able to propose the optimal level of medium compositions to achieve the most appropriate results of the investigated parameters. The optimized medium compositions via RBF-NSGAII resulted in 100% PR, 12.87 SN, 4.63 cm SL, and 0.06 g BCW (Table 5). The results indicated that the difference between the MLP predicted and validation data was negligible (Table 5).

**Table 5:** Validation of the predicted data for proliferation rate (PR), shoot number (SN), shoot length (SL), and basal callus weight (BCW) of chrysanthemum in validation experiment.

Treatment	PR (%)	SN	SL (cm)	BCW (g)
Ideal point in NSGAII process	100 ± 0.00	12.87 ± 0.15	4.63 ± 0.13	0.06 ± 0.02

Values in each column represent means ± SE.

## Discussion

Being successful in plant tissue culture depends on various factors such as gelling agents, the composition of the medium, use of specific combinations of PGRs, and light and temperature conditions^2^. Adjusting medium compositions for increasing plant growth and development is the most common method in plant tissue culture^2,9^. In the current study, RBF-NSGAII model was used to achieve a comprehensive understanding of the effect of different levels of BAP, IBA, PG and sucrose on shoot proliferation of chrysanthemum, and to obtain new insights into improving chrysanthemum organogenesis. According to the best of our knowledge, this study is the first report of using RBF-NSGAII for modeling and optimizing medium compositions for shoot proliferation of this ornamental plant.

High coefficient of determination between observed and predicted values for both training and testing process indicated good performance of the models for the studied parameters. The high efficiency of ANN in plant tissue culture has been shown by several studies^26,27,30^. Recently, several studies used ANN-GA for modeling and optimizing *in vitro* organogenesis^26,27^. GA is mainly used for optimizing different *in vitro* conditions using a single objective function. However, plant tissue culture problems have to satisfy various objective functions by considering different constraints and GA, as a single-objective algorithm, cannot optimize multi-objective simultaneously^37^. Therefore, the use of GA would not be the applicable solution to optimize multiple-objective functions. Thus, there is a dire need for applying the multi-objective algorithm for the optimization process. In this study, we have introduced NSGAII as a multiple-objective algorithm. BCW has a negative effect on micropropagation due to the somaclonal variation and restriction of vascular system. Therefore, the ultimate aim of this study was to analyze RBF model to provide an accurate answer of what levels of medium compositions may be applied to obtain the maximum PR, SN, and SL as well as the minimum BCW.

Shoot proliferation usually produces true clones of an explant. In some plants such as chrysanthemum, it is not feasible to achieve micro-shoots by using PGRs or manipulation culture-room conditions^2,9^. Adjusting medium composition as an alternative for the promotion of shoot proliferation would benefit *in vitro* culture^19^. Several studies^15,39–41^ reported the successfulness of shoot proliferation of chrysanthemum via single node explants. However, those studies focused on the effects of different hormonal combinations on shoot proliferation. Therefore, there is a lack of a comprehensive study on the effect of medium composition in shoot proliferation. The results of this study showed the necessity of balancing cytokinin/auxin (BAP/IBA) for shoot regeneration. Similarly to our results, Iizuka, *et al*.^42^ showed the maximum shoot regeneration in MS medium supplemented with 8.88 µM BAP and 0.1 µM IBA. Also, Lu, *et al*.^41^ reported that the combination of 8.88 µM BAP and 1.07 µM NAA resulted in 100% PR in chrysanthemum. It is well known that *in vitro* organogenesis significantly depends on the ratio between cytokinins and auxins^12,13^. The previous studies indicated that the high ratio of cytokinin/auxin promote shoot induction^9,13,14,41,42^.

In our experiment higher PR, SN and SL were achieved on media with supplemented PG, however PG concentrations over 0.3 mM had an inhibitory effect on these parameters. A recent study^23^ reported that PG could promote shoot regeneration in *Vitex negundo*. Additionally several studies^19,23,24^ associated PG with plant growth and development. Shoot proliferation of *Minuartia valentina* was improved on MS medium with PG in combination with BAP^43^. Sarkar and Naik^22^ indicated PG × sucrose interaction plays an important role in shoot regeneration. Similarly, our results showed that 0.29 mM PG and 87.63 mM sucrose had a striking impact on shoot proliferation.

According to our results, 87.64 mM sucrose was better during the shoot proliferation stage, which was consistent with the findings of Lu, *et al*.^41^, da Silva^2^, Iizuka, *et al*.^42^, and Arun, *et al*.^44^ who suggested that 87.64 mM sucrose should be used in all culture stages for the *in vitro* multiplication of chrysanthemum.

Finally, according to the validation experiment, RBF-NSGAII can be considered as a new computational algorithm in analyzing data derived from *in vitro* culture parameters for predicting optimized levels of medium compositions required in the shoot proliferation stage.

## Conclusion

*In vitro* culture issues have to satisfy different opposite objective functions; so, there is a dire need of using the multi-objective algorithms such as NSGA-II for optimizing the process. RBF-NSGAII has been introduced as an applicable algorithm for modeling and optimizing shoot proliferation of chrysanthemum. Based on the results of this study, the interaction effects of medium compositions can be precisely identified via RBF-NSGAII. Generally, RBF-NSGAII can be considered as a powerful and applicable model for applying in various areas of plant tissue culture.

## Methods

### Plant materials

The single-node explants of chrysanthemum ‘Hornbill Dark’ were cut from indoor mother plants. After that, the explants were washed for 30 min under tap water, followed by washing after cleaning by a liquid soap solution. Additional surface sterilization was implemented under a laminar airflow chamber. The explants were sterilized by 70% aqueous ethanol for 40 s, dipped 15 min in 1.5% (v/v) NaOCl solution, and three times washed by sterilized distilled water. Then, the nodal segments (0.5 cm) were vertically inoculated on 200-ml glass flasks consisting of 40 ml basal medium.

### Media and culture condition

MS^45^ medium supplemented with 0.7% agar (Duchefa Biochemie, Netherlands) and 6% glucose was used as a basal medium in this experiment. In addition, the pH of the medium was set to 5.8 through applying 1 N HCl or 1 N KOH before 20 min autoclaving at 121 °C. All culture vessels were incubated at 26 ± 2 °C under a 16-h photoperiod with the light intensity of 60 µmol m^−2^ s^−1^.

### Experimental design

The experiments were set up based on the completely randomized design (CRD) with the factorial arrangement in five replicates and nine sets of repetitions per treatment.

The explants were cultured in the proliferation medium containing different concentrations of BAP (0, 2.22, and 4.44 μM), IBA (0, 0.25, and 0.49 μM), PG (0, 0.2, and 0.4 mM) and sucrose (43.82, 87.64, and 131.46 mM). The PR, SN, SL, and BCW were determined after 8 weeks of culture.

### Radial basis function artificial neural network (RBF-ANN)

**Figure 5:**
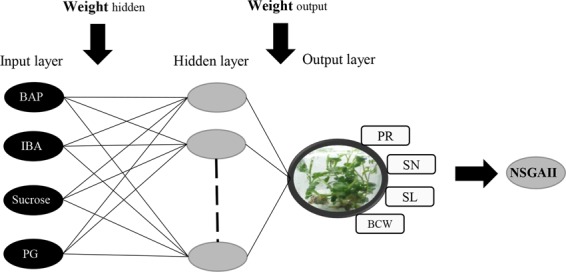
The schematic diagram of the proposed RBF methodology.

In order to model medium composition BAP, IBA, PG and sucrose were used as inputs while PR, SN, SL, and BCW were considered as outputs (Fig. 5). The algorithm chosen to model these relationships was RBF-ANN. RBF is a statistical neural network used for regression-based problems. The input of the transfer function for each neuron in such a network is the Euclidean distance between the input and the center of that neuron^46^. RBF was applied for obtaining the maximum rate of PR, SN, and SL as well as the minimum rate of BCW. Prior to modeling the data was split into a 75% training and 25% testing set and checked to confirm that the ranges of the train and test data are overlapping. The different values for the significant model’s parameters were examined based on a trial and error analysis for determining and improving the overall performance of the best-constructed model. For trial and error analysis, we used MSE as the default criteria for tuning ANN models. In addition, we used K-Fold Cross Validation (K = 5) for our training set. Also, we trained four RBF models for each outputs including PR, SN, SL, and BCW. In the end, the best-resulted output with the minimum estimation error was determined for each individual model based on Mean Bias Error (MBE), Root Mean Square Error (RMSE), and the coefficient of determination (R2) as follows:

1 $${R}^{2}=1-\frac{{\sum }_{i=1}^{n}{({y}_{i}-{\hat{y}}_{i})}^{2}}{{\sum }_{i=1}^{n}{({y}_{i}-{\bar{y}}_{i})}^{2}}\,(0\le {{\rm{R}}}^{2}\le 1)$$

2 $$RMSE=\sqrt{({\sum }_{i=1}^{n}{({y}_{i}-{\hat{y}}_{i})}^{2})/n}\,(0\le {\rm{RMSE}}\le \infty )$$

3 $$MBE=1/n\,{\sum }_{i=1}^{n}|{y}_{i}-{\hat{y}}_{i}|\,(\,-\,1\le {\rm{MBE}}\le +1)$$

where ${y}_{i}$ is the value of predicted datasets, ${\hat{y}}_{i}$ is the value of observed datasets, and n is the number of data. Best fit can be achieved when R^2^ values closer to 1 and RMSE values closer to 0. The MBE value stands for negative and positive calculation error, indicating the similarity of the predicted values with observational values.

Popular transfer function in RBF is the Gaussian function, the Gaussian function uses the following relation^46^:

4 $$f({X}_{r},{X}_{b})={e}^{-{[\Vert {X}_{r}-{X}_{b}\Vert \ast 0.8326/h]}^{2}}$$

where ${X}_{r}$: input with unknown output, ${X}_{b}$: observed inputs in time *b*, and h: spread. The output of the function close to 0 when $\Vert {X}_{r}-{X}_{b}\Vert$ approaches a large value, and close to 1 when $\Vert {X}_{r}-{X}_{b}\Vert$ approaches 0. Eventually, the dependent variable (*Yr*) by predictor *X*_*r*_ is calculated as follows:

5 $${Y}_{r}=\mathop{\sum }\limits_{b=1}^{m}{w}_{b}\,\ast \,f({X}_{r},{X}_{b})+{w}_{0}$$

where ${w}_{j}$ is the weight of connections from the *b*^th^ hidden layer to the output layer and *w*_0_: bias.

### Optimization process (NSGAII)

**Figure 6:**
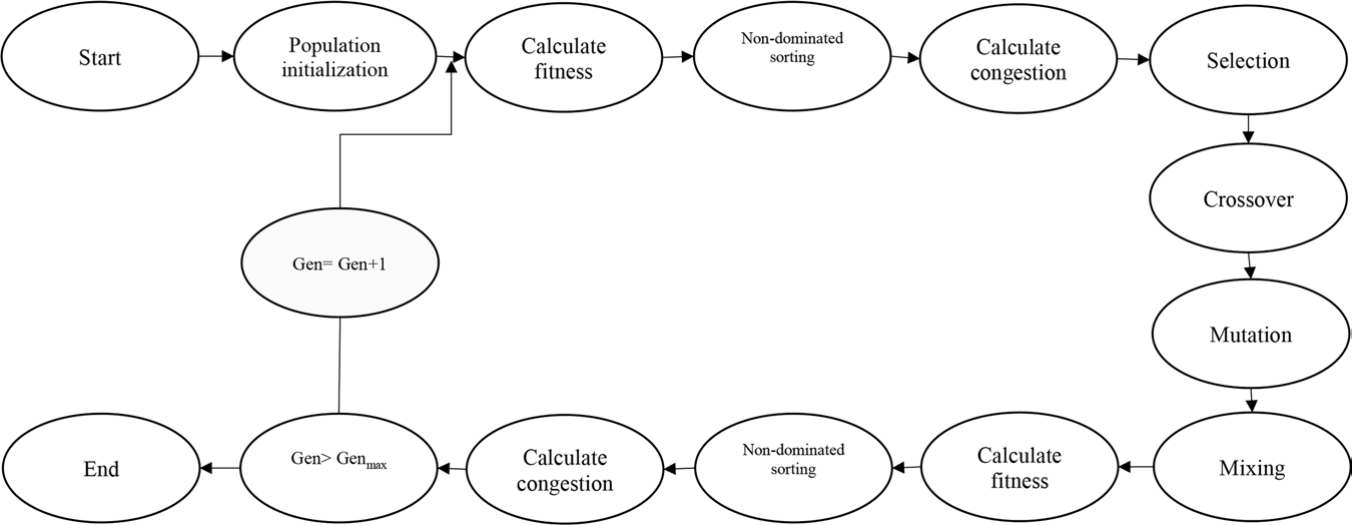
Schematic diagram showing the step-by-step NSGAII optimization process.

NSGA-II is an evolutionary optimization algorithm that is used in multi-objective problems. This algorithm starts by generating a set of random solutions; the objective function value is then calculated for each solution, and the process of refining the solutions begins. At this step, the solutions are elected for crossover using the binary tournament operator based on two criteria: non-dominated sorting and crowding distance. The algorithm can be kept from getting stuck in the local optimum by applying a mutation operator. The objective function values are calculated once again after the refining solutions are determined. This process is repeated until one of the stopping criteria is satisfied. In each generation, non-dominated solutions in objective space constitute a pareto front; any point on this front can be an optimal solution of the problem (Fig. 6). In this study, 200 initial population, 1000 generation, 0.7 crossover rate, 0.05 mutation rate, the uniform of mutation function, two-point crossover function, and a binary tournament selection function were considered. Also, PR, SN, SL, and BCW were considered as four objective functions to determine the optimum values of inputs based on the results of RBF algorithm. The ideal point of pareto was chosen such that PR, SN, and SL were maximized, while BCW was minimized. In other words, a point in the pareto front was considered as the solution such that

6 $$\sqrt{{(PR-m)}^{2}+{(SN-n)}^{2}+{(SL-o)}^{2}+{(BCW-p)}^{2}}$$

was minimal; where *m*, *n*, and *o* are the maximum PR, SN, and SL respectively, and *p* is the minimum BCW in observed data. Before applying Eq. 6, objective function values were scaled between 0 and 1.

### Sensitivity analyses

The sensitivity PR, SN, SL, and BCW against the investigated medium compositions, was assessed by considering the following criterion;

Sensitivity error (VSE) value: overall performance of the developed RBF model if the certain independent variable is not available.

Variable sensitivity ratio (VSR) value: indicates the correlation between the VSE and the error of the RBF model in the case that all variables are available.

High rate of VSR is paramount. Therefore, all input variables should be ranked based on their VSR rate (importance). The mathematical code for constructing and assessing models and optimization analysis was written conveniently for Matlab (version 9.5) software.

### Validation experiment

During the validation experiment, the levels of medium compositions optimized by RBF-NSGAII were evaluated for confirming the efficiency of RBF-NSGAII to model and optimize the medium composition for shoot proliferation parameters (i.e., PR, SN, SL, and BCW).
